# *In vivo* evidence of angiogenesis inhibition by β_2_-glycoprotein I subfractions in the chorioallantoic membrane of chicken embryos

**DOI:** 10.1590/1414-431X202010291

**Published:** 2021-01-15

**Authors:** C.M. Baldavira, L.F. Gomes, L.T. De La Cruz, D.A. Maria, V.L. Capelozzi

**Affiliations:** 1 Universidade de São Paulo, Departamento de Patologia, Faculdade de Medicina, São PauloSP Brasil Departamento de Patologia, Faculdade de Medicina, Universidade de São Paulo, São Paulo, SP, Brasil; 2 Universidade de São Paulo, Departamento de Análises Clínicas e Toxicológicas, Faculdade de Ciências Farmacêuticas, São PauloSP Brasil Departamento de Análises Clínicas e Toxicológicas, Faculdade de Ciências Farmacêuticas, Universidade de São Paulo, São Paulo, SP, Brasil; 3 Universidade de São Paulo, Laboratório de Sistemas Planctônicos, Instituto Oceanográfico, São PauloSP Brasil Laboratório de Sistemas Planctônicos, Instituto Oceanográfico, Universidade de São Paulo, São Paulo, SP, Brasil; 4 Instituto Butantan, Laboratório de Biologia Molecular, São PauloSP Brasil Laboratório de Biologia Molecular, Instituto Butantan, São Paulo, SP, Brasil

**Keywords:** Angiogenesis, Chicken embryo, Chorioallantoic membrane, Morphometry, Beta-2-glycoprotein I

## Abstract

The vascular network expansion and functioning are important factors affecting normal intra-uterine fetal development. This study addressed the previously reported antiangiogenic potential of beta-2-glycoprotein I (β_2_GPI) *in vivo* in the chick embryo model of angiogenesis. The effects of two naturally occurring β_2_GPI forms on the development of the chorioallantoic membrane (CAM) vessels and the chicken embryo were investigated. β_2_GPI monomers and dimers were obtained by fractioned purification and characterized using SDS-PAGE, immunoblot, and ELISA. The egg exposure was performed by injection of small volumes of 2.5 µg/mL solutions of the β_2_GPI subfractions. Angiogenesis was evaluated through quantitative measurements of vascular architecture parameters in the captured CAM images, using computational analysis of texture contrasts and computer vision techniques. Quantitative information was assigned to the CAM vasculature modifications. *In vivo*, the β_2_GPI dimer completely halted the formation of CAM vessels and led to embryo death after 48 h of exposure. The β_2_GPI monomer allowed the embryo to develop up to the 10th day, despite early changes of CAM vessels. The impaired normal vessel growth proceeded as a self-limited effect. The β_2_GPI monomer-exposed eggs showed reduced vascularization on the 6th day of incubation, but embryos were viable on the 10th day of incubation, with ingurgitated CAM vessels implying sequelae of the angiogenesis inhibition. Both subfractions impaired CAM vasculature development. The β_2_GPI dimer proved to be largely more harmful than the β_2_GPI monomer. β_2_GPI modification by cleavage or dimerization may play a role in angiogenesis control *in vivo*.

## Introduction

Some of the most important factors that affect normal intra-uterine development in fetuses are the processes regulating the origin and proliferation of the vascular network, namely vasculogenesis, and angiogenesis. Vasculogenesis is the production of vessels from angioblasts, whereas angiogenesis involves branching and developing new vasculature from pre-existing vessels. Both processes are critical to embryo growth. The inhibition of angiogenesis during pregnancy is associated with a higher risk of gestational abnormalities, including congenital malformations, histopathological injuries, and growth retardation (1,2). Numerous pathways, growth factors, and proteins are critical for the regulation, promotion, and inhibition of these processes.

One of the proteins under investigation for its effects on vascular development is the plasma protein beta-2-glycoprotein I (β_2_GPI), which has recently been recognized for its anti-angiogenic properties both *in vitro* and *in vivo* (3–5). Human β_2_GPI is a single-chain glycoprotein found in the plasma, with five short consensus repeats domain sequences (domains I-V) typical of the complement control protein superfamily proteins (6). The fifth domain (DV) differs from D I-IV in that it includes a lysine-rich positively charged region and an extra 20-amino acid sequence, in a movable tail chain that projects from the main surface of β_2_GPI, both features being crucial for protein-protein interactions and phospholipid binding (6). The β_2_GPI gene is conserved across the animal kingdom, with homology among the human, chimpanzee, dog, cow, mouse, rat, and chicken (7).

The β_2_GPI molecule is known as the major antigen in the anti-phospholipid syndrome (6), an auto-immune disease characterized by thrombosis and recurrent abortions. Although the precise physiological function of β_2_GPI remains undefined, researchers have uncovered its reverse effects on many components of the coagulation and fibrinolytic cascade (8). Specifically, β_2_GPI interacts with plasmin in the plasminogen/plasmin cascade (5,6), leading to the cleavage of β_2_GPI by proteolysis in the 20-amino acid tail of DV, thus creating a “nicked” β_2_GPI that binds to plasminogen and inhibits plasmin production, promoting an anti-angiogenic effect through inhibition of both fibrin degradation and matrix metalloproteinase activation. The nicked β_2_GPI also shares a common binding site in annexin 2 with angiostatin and plasmin, providing an attenuation pathway for modulation of its anti-angiogenic function (5).

The complex effect of β_2_GPI on both angiogenesis and vasculogenesis was previously addressed *in vitro*. Regarding *in vivo* models for angiogenesis and vasculogenesis, chick embryos provide a suitable preclinical model due to their simplicity, low cost, reproducibility, and worldwide consensus about its ethical and legal aspects (9). The vascular plexus of a chick's chorioallantoic membrane (CAM) is simple and branches progressively during embryonic growth. It is additionally suitable for research on the concurrent prothrombotic and teratogenic effects of β_2_GPI while modeling the congenital adverse effects of abnormal vascular growth on fetal development during pregnancy (10).

Native β_2_GPI monomers and dimers were demonstrated to induce differential effects on the proliferation and differentiation of endothelial cells in two-dimensional cultures used as an angiogenesis model (11). It was not elucidated if the mechanism of these β_2_GPI effects on angiogenesis depends on the dimer formation.

Therefore, the development of CAM vessels was used to address embryonic vasculogenesis and angiogenesis effects of β_2_GPI *in vivo*. The vascular branching patterns in the developing CAMs exposed to β_2_GPI were evaluated, quantified by automated computer vision algorithms, and associated with early embryo development events.

## Material and Methods

### β_2_GPI purification fractions

Human β_2_GPI was affinity-purified from long-term stored human plasma to benefit from spontaneous β_2_GPI dimerization. Individual plasma bags from at least three different donors were kept under -80°C for three to five years, defrosted at 4°C, pooled, and purified by affinity chromatography in a Heparin-Sepharose column (Heparin Sepharose 6 Fast Flow, GE Healthcare, Brazil), as described by Polz et al. (12). Dimer and monomer rich aliquots were identified using a 12.5% SDS-PAGE electrophoresis, assayed as independent purified fractions, and used without further processing. Purity was estimated by the potential binding to negatively charged phospholipids using ELISA (13). The monomeric and dimeric fractions were isolated and dialyzed against deionized ultrapure water (Milli-Q, Merck Millipore, USA) (13).

### *In vivo* angiogenesis assay

The eggs were incubated as described by Ribatti et al. (14) in a brooder (Zagas, Brazil) at 51% internal humidity and 37°C. On the 4th day of incubation, the eggs had part of their albumin removed (500 µL) gently and slowly, and, posteriorly, were exposed to β_2_GPI by careful and slow injections of 200 µL of the stock solutions (12.5 µg/mL) of the monomer or the dimer subfractions diluted in egg albumin before injection (to produce a 2.5 µg/egg/dose). The control was treated with albumin diluted in pure water. The 2.5 µg/egg per dose was selected for this study because independent tests on endothelial cells (HUVECs) demonstrated that it did not produce mitochondrial toxicity (11).

The viability of the embryos was monitored by ovoscopy throughout the incubation period. Embryo death in the dimer-exposed group served as the criterion for test interruption, typically on the 6th day of incubation. Monomer-exposed and respective controls could be incubated until the 10th day. The sampling procedures were carried out after embryonic hypothermia, as described by Pereira-Lopes et al. (15). Embryos were weighed and measured immediately after sampling, and images were registered. CAMs were then removed, rapidly fixed in diluted buffered formaldehyde, and analyzed while still wet on a glass surface using a low magnification stereomicroscope (Nikon SMZ1800, Japan; 2× magnification) or scanner (Hewlett Packard Scanjet G2410 Flatbed Scanner, Brazil) to obtain their images (1200 dpi resolution scanner under high-resolution settings).

This study was carried out in strict accordance with the recommendations of the Guide for the Care and Use of Laboratory Animals of the National Institutes of Health. The protocol was approved by the Committee on the Ethics of Animal Experiments of the Faculty of Medicine, University of São Paulo (Permit Number: 529/13), and the Committee on the Ethics of Animal Experiments of the Butantan Institute (Permit Number: 1124/13). All efforts were made to minimize suffering.

### Computational analysis

The captured CAM images were submitted to a texture analysis using computer vision techniques for quantitative evaluation of vasculature parameters. This analysis was performed as described by Ojala et al. (16) through uniform and rotation invariant Local Binary Patterns (LBP). In short, the LBP approach consists of classifying each pixel in the image according to the relationship between the intensity of each pixel and that of its neighbors (16,17). In the present study, LBP was determined using software coded in Matlab R2013 (Mathworks, USA) designed to process the CAM images. The result of the texture analysis was then either described in histograms that quantitatively represent the textures in each image or represented in new color images that visually demonstrate their distribution. The spatial distribution of the patterns allowed our team to verify the correspondence between the assigned patterns and the structures of interest in the original image. The comparison was made using 1024×1024 squared pixel fields captured from each of the samples. Significant differences between controls and treatments were determined with the Kruskal-Wallis test using MATLAB^®^ software R2017a. The minimum acceptable confidence level was set at 95% (P<0.05).

## Results

### Embryo size and weight

**Figure 1:**
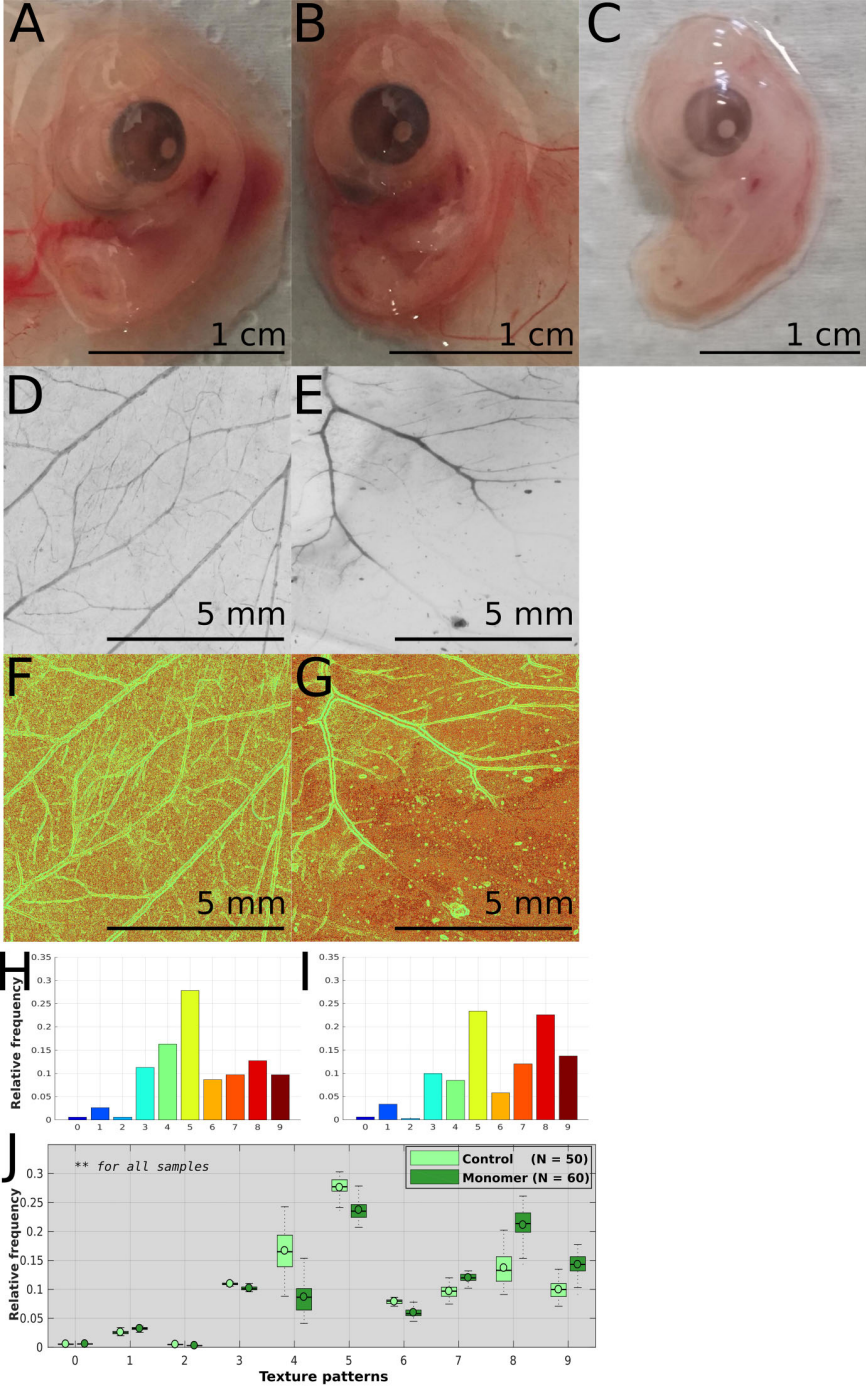
Embryos and chorioallantoic membrane (CAM) morphology data and results as obtained on the 6th day of incubation. **A**-**C**, Macroscopic aspect of the embryos (scale bars: 1 cm). **D** and **E**, Scanner images of the CAM (scale bars: 5 mm). **F** and **G**, Typical quantitative image result obtained after individual Local Binary Patterns image processing (scale bars: 5 mm) and **H** and **I**, corresponding histogram. **J**, Comparative distribution of the CAM results of unexposed controls and embryos exposed to β_2_GPI monomer (2.5 µg/egg). All samples presented **P<0.01 (the value of N represents the number of fields used in the analysis). The CAM of embryos exposed to β_2_GPI dimer (2.5 µg/egg) (**C**) could not be analyzed. Images were taken 48 h after the exposure to β_2_GPI purification fractions and vehicle, or vehicle only in their respective controls. Images **A**-**C** were taken with Canon Powershot G12 equipment. Images **D**-**E** were taken with a 1200 dpi resolution scanner under transillumination and high-resolution settings. Significant differences between controls and treatments were determined with the Kruskal-Wallis test using MATLAB^®^ software R2017a.

Table 1 shows the size and weight of the embryos exposed to β_2_GPI, on the 6th and the 10th day of incubation. The β_2_GPI monomer treatment proved compatible with the development of viable embryos beyond this period. The interruption of embryo development in the β_2_GPI monomer and control groups on the 6th day of incubation was carried out to investigate the morphology of these embryos under the same incubation time and conditions in which embryos exposed to β_2_GPI dimer died. Exposure to the β_2_GPI dimer, on the 4th day of incubation *in vivo*, induced early embryonic death of all embryos in this group, on the 6th day of incubation (Figure 1C; Table 1).

**Table 1:** Effects of the β_2_GPI subfractions on embryo development.

	6th day			10th day		
	Head-tail length (cm)	Weight (g)	N	Head-tail length (cm)	Weight (g)	N
Control	1.9±0.1	0.36±0.05	10	3.6±0.1	1.66±0.13	2
Monomer	1.8±0.2	0.41±0.07	6	3.4±0.1	1.96±0.14	3
Dimer	2.3±0.1	0.44±0.08	5	-	-	-

Data are reported as mean±SD.

**Figure 2:**
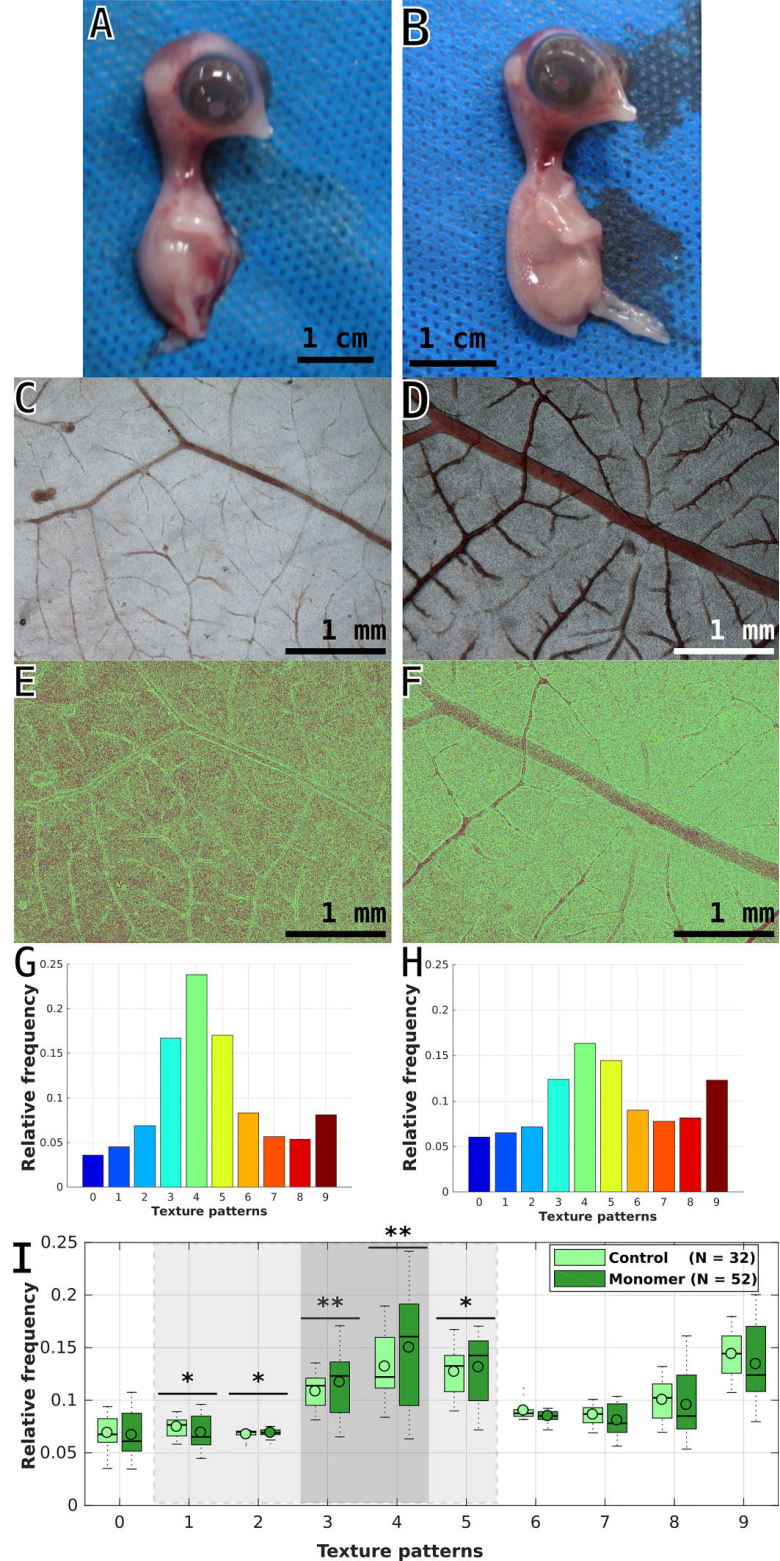
Embryos and chorioallantoic membrane (CAM) morphology data and results obtained on the 10th day of incubation. **A** and **B**, Macroscopic aspect of the embryos (scale bars: 1 cm). **C** and **D**, Stereomicroscope images of the CAM vessels (scale bars: 1 mm). **E** and **F**, Typical quantitative image result obtained after individual LBP image processing (scale bars: 1 mm) and **G** and **H**, corresponding histogram. **I**, Comparative distribution of the CAM results of unexposed controls and embryos exposed to β_2_GPI monomer (2.5 µg/egg), horizontal bars represent statistical differences between groups (*P<0.05 (light gray); **P<0.01 (dark gray)); the value of N represents the number of fields used in the analysis. Images were taken 144 h after exposure to β_2_GPI monomer and vehicle, or vehicle only in their respective controls. Images **A** and **B** were taken with a Canon Powershot G12 equipment. Images **C** and **D** were taken with a digital DS-U1 camera attached to a stereoscopic Nikon SMZ1800 microscope under 2× magnification, standardized illumination, shadow regulation, and TIFF image resolution, with the help of Nikon software ACT-2U. Significant differences between controls and treatments were determined with the Kruskal-Wallis test using MATLAB^®^ software R2017a.

The size of the monomer-treated embryos was similar to that of the control embryos on both the 6th and the 10th days of incubation (Figures 1 and 2; Table 1). However, the dimer group embryos showed an edematous appearance compatible with stress induction and reduced development at the end of the 6th day of incubation (Figure 1; Table 1). The size and weight of the treated and the control group embryos were not different (Table 1; P>0.05).

### Vascular branching pattern

Figure 1 shows the CAM images captured on a scanner on day 6 (Figure 1D and E), as well as the results of their computational analysis (Figure 1F and G). CAM images were obtained from the embryos of the control (Figure 1D and F) and β_2_GPI monomer (Figure 1E and G) groups. No vascular network was formed in the CAMs of the embryos treated with the β_2_GPI dimeric fraction of the protein. In this case, CAM vessels either failed to develop or regressed, and the vessels of the yolk sac also regressed, being, in some cases, nearly absent. The CAM images of the 6th day of incubation, obtained with an ultrasensitive scanner, revealed early structural alterations in the vascular network, induced by the β_2_GPI monomer. While the control eggs exhibited an initial organization of the hierarchical structures compatible with this developmental stage (Figure 1D and F), the β_2_GPI monomer-exposed embryos presented less branched and structurally anomalous vasculature (Figure 1E and G).

Figure 1 H-J illustrates the profile of the frequencies of the calculated LBP values generated from the CAM images captured on day 6. Figure 1H shows the typical aspect of a control embryo LBP profile frequencies distribution. Figure 1I shows the correspondent profile distribution for a β_2_GPI monomer exposed embryo. The LBP profiles revealed quantitative differences in their distribution between the two groups, while LBP profile frequencies distribution was relatively homogeneous within each experimental group (Figure 1J). The uniform patterns associated with border and corner elements were proportionally the most abundant among the controls (Figure 1F and H). Conversely, the patterns associated with the vessels' borders were as abundant as those associated with the vessels' lumen and avascular regions among the embryos exposed to the β_2_GPI monomer (Figure 1G and I). This result quantitatively represents the dissimilarities in the microcirculation mesh and the complexity of the CAM vasculature that distinguishes the branching and growth of the vascular trees of the experimental groups. Vascular and linear structures associated to the LBP profile number 4 are expected to be the most abundant. However, the distribution of LBP profiles numbers 3, 5, and 6 depends on the prevalence of many delicate, less congested, and ramified vascular tree elements that were more prevalent among the controls than among the exposed embryos. The lower frequencies of the patterns associated with the branched and less regular vascular structures revealed either the loss of small vessels mesh or an asynchronously developed CAM vasculature among the exposed eggs, in which the primitive blood islands remained, and the small twisted arranges of budding vessels were less observable. The corresponding increase of vessel lumen and background associated patterns were observed among monomer group CAMs. Statistical differences were shown for all LBP profiles analyzed.

Figure 2 shows the CAM images captured on day 10 from the control (Figure 2C and E) and β_2_GPI monomer (Figure 2D and F) groups. When analyzed under a stereoscopic magnifying glass, the CAMs of the control eggs presented organized vessels in the usual pattern of branching, with a predominantly symmetrical distribution, respecting the hierarchical pattern of the sequential vascular branches concerning the vessel caliber and the distance between the branches. Typically, there were between three and six sequential branches between the thinnest and the thickest vessels of the membrane (Figure 2C and E). In contrast, the embryos exposed to the β_2_GPI monomer showed CAM vessels hierarchy with fewer sequential branches. β_2_GPI monomer treatment also elicited the development of a large number of vessels with either long unbranched segments or segments presenting asymmetric forks and occasional parallel vessels. We were able to observe atypical congestion of the main percolation pathways and larger vessels, as well as the presence of triangle-shaped forks, reduced vascular budding, and vascular anastomoses (Figure 2D and F).

The profile of the frequencies of the LBP values generated from the images captured on day 10 is shown in Figure 2. The intragroup dispersion or the LBP relative frequencies observed for the control samples was smaller than that observed for the β_2_GPI exposed samples for all LBPs (Figure 2I). The quantitative effects dissimilarities were more pronounced among the samples collected on the 6th day of incubation than among those collected on the 10th day (Figures 1 and 2). On the 10th day of incubation, the β_2_GPI exposure effects on the CAM texture patterns were associated to the production of larger and congested vessels connected to a less dense capillary network, contrasting with the control group, which exhibited highly interconnected and branched vessel trees, with a higher density of partially filled small vessels interspersed within the larger vessels. The statistical differences are shown for the LBP profiles associated with vessel borders, branching corners, and terminal segments.

## Discussion

The differential angiogenic potential of both the monomeric and dimeric β_2_GPI purified subfractions, as addressed by the CAM model in this work, sheds light on the *in vivo* effects of β_2_GPI. The present results may contribute not only to future studies on gestational abnormalities but also to a deeper understanding of vascular growth and endothelial regeneration impairment as an intercurrence in the pathophysiology of autoimmune and infectious diseases (9,18).

Regarding mechanistic aspects, β_2_GPI putative receptors have been reported to be expressed in endothelial cells, being activated by specific antibodies to express adhesion molecules and tissue factors (19) and share biochemical routes relevant to the physiology and development of the cardiovascular system (20,21). Also, β_2_GPI was reported to increase the expression of anti-angiogenic factors such as platelet factor-4 (13), angiostatin, thrombospondin-1, and others (22). Our findings suggested that β_2_GPI impaired angiogenesis *in vivo*, though its monomeric fraction had less of an impact than the dimeric fraction. The major angiogenesis-related receptors, vascular endothelial growth factor (VEGF) and basic fibroblast growth factor (bFGF), are not β_2_GPI ligands (4) and previous research showed no VEGF-R1 inhibition promoted by β_2_GPI. However, the protein is known to reduce the expression of VEGF-R2 and dose-dependently inhibit both the formation of tubes on Matrigel and the proliferation of HUVECs induced by VEGF or bFGF in 2-D cultures. The nicked form of the protein inhibits the phosphorylation of Akt and ERK1/2 (4). Accordingly, β_2_GPI seems to regulate the growth and directional migration of human aortic endothelial cells (HAEC) and inhibit the VEGF-induced phosphorylation of VEGF-R2, ERK1/2, Akt, and eNOS (23). Both the native and nicked forms of the protein show inhibitory effects on the proliferation and differentiation of HUVECs (4,24). The nicked form is the most active in protein-associated effects such as the modification of the phosphorylation of ERK1/2, JNK, and p38, and promotion of the expression of effector proteins and cell cycle controllers in EOMA cells (25). In vascular endothelial cells of the choroid-retinal retina, β_2_GPI inhibited angiogenesis when induced by advanced glycation end products, thus reducing the expression of VEGF-R2 and its effector molecules on the MAPK, ERK1/2, and PI3K/Akt pathways (26).

Annexin-2 has been described as a functional ligand to β_2_GPI at the endothelial cell surface (25), and it was demonstrated as a capillary endothelial marker of the chorionic layer throughout embryonic development (27). The involution of CAM vasculature as observed in this work was compatible with the literature reported annexin-2 mediation of the β_2_GPI effects in vivo. Furthermore, annexin-2 binding on the endothelial cell surface was reported to induce apoptosis in endothelial cells and regression of angiogenesis (28). Similarly, changes in annexin-2 lead to changes in signaling through the Akt pathway (29). The Notch pathway was proposed as a new target for the β_2_GPI effects *in vivo*. This pathway determines the formation of functional vessels during embryonic angiogenesis and in several models of tumor angiogenesis. Among the transmembrane signaling molecules that participate in this signaling, Dll4 expression is precocious during vasculogenesis, is the first Notch ligand expressed in the arterial endothelium (30), and its expression is induced by VEGF (31).

Thus, we highlight the correspondence between the present results and the effects of β_2_GPI previously observed *in vitro* (12). The observed impairment of vasculogenesis and angiogenesis and the abnormality of embryonic development, including the occurrence of early lethality, are compatible with inhibition in Akt and Notch.

The present results with the CAM model revealed that structural anomalies related to losses of complexity and symmetry of the vascular network, corresponding either to interruption of development or early induction of vascular regression, may depend on the composition of β_2_GPI subfractions. Then, the β_2_GPI inactivation by cleavage, induced by inflammation or autoimmunity, may play a role in angiogenesis control through dimerization and differential binding to putative β_2_GPI receptors and active signaling cofactors like annexin-2, ApoER2, Toll-like receptor 4, structural negative polysaccharides, and MHC class II molecules (6).

Early embryonic vasculogenesis and angiogenesis in the CAM of chick embryos were assessed using an automated computer vision algorithm. The *in vivo* evidence showing how the β_2_GPI subfractions exert divergent effects on chick embryos and extraembryonic tissues included: 1) embryos exposed to the β_2_GPI dimer either experienced early embryonic death or presented as amorphous and almost avascular masses within 48 h after exposure, whereas all of the embryos exposed to the β_2_GPI monomeric fraction remained alive after this time; 2) exposure to the β_2_GPI monomeric fraction allowed the chick embryos to develop until at least the 10th day of incubation, while the β_2_GPI dimeric fraction induced early changes in the development of the vessels of the CAM, as well as embryo death on the 6th day of incubation; and 3) CAM vessels either failed to develop or regressed in the embryos treated with the β_2_GPI dimeric fraction, while the β_2_GPI monomer-exposed embryos were only mildly affected, presenting less branched vasculature.

The LBP texture histograms quantitatively represented the dissimilarities in the CAM microcirculation mesh, revealing the complexity of the CAM vessels architecture that could distinguish between the effects of β_2_GPI subfractions. The dramatic effect of the dimeric fraction emerges as additional evidence of the relevance of ancient mechanisms in maintaining physiological control over β_2_GPI oxidation and dimerization *in vivo* (32). Both the oxidation of β_2_GPI by endothelial cells *in vitro* and the antioxidant properties of the protein's monomeric fraction have been described in previous studies (33). Other complex metabolic effects have been previously addressed using the chick embryo pre-clinical model (9) that are suitable to further investigate the effects of β_2_GPI *in vivo*. Most *in vitro* findings on the effects of β_2_GPI subfractions on angiogenesis come from endothelial cell lineages (20). Although *in vitro* behavior of endothelial cells often differs from that observed in the *in vivo* microenvironments, and immortalized endothelial cells may undergo functional changes distinct from that of native cells, the extent of the β_2_GPI binding to the cell surface seemed to depend on the different endothelial cell origins within the vascular tree (34). Divergent effects on hemostasis were also described for the β_2_GPI subfractions; while the dimeric fraction showed pro-thrombotic properties, the monomeric fraction had anti-thrombotic effects.

Protein cleavage and dimerization after inflammatory activation may have a regulatory effect on vessel growth and endothelium-dependent vascular maturation, as previously suggested by the results obtained with a simpler angiogenesis model *in vitro* (11). Even if the inhibitory effect of β_2_GPI on the migration of endothelial cells *in vitro* was not implied with reduction of cell proliferation or survival (23), the nicked forms, the protein dimers, and the aggregates may play an inhibitory effect on other processes, as suggested by the pathophysiology of the thromboembolic diseases in which these protein-protein interaction forms are produced *in vivo*. There are several reports on the effect of protein on angiogenesis, both *in vitro* and *in vivo* (3,5,11,23,25). Neither approach allowed a single target or specific pathway to be assigned to the anti-angiogenic effects of the protein.

Although it is possible that the protein targets both the vasculogenesis and the angiogenesis processes through effects on endothelial cell migration and VEGF-dependent growth, one could argue that functional differences of β_2_GPI subfractions should be detectable using this *in vivo* angiogenesis model (20), whereas endothelial cells migration and VEGF-induced neovascularization inhibition by β_2_GPI was previously reported (23). Antiangiogenic properties were previously ascribed to plasma β_2_GPI regardless of its subfraction composition (3–5). This antiangiogenic effect allowed β_2_GPI to inhibit the growth of tumor implants in mouse models (35).

The β_2_GPI monomer is the fraction that predominates physiologically *in vivo*, but in biological fluids, this protein is mainly associated with negative surfaces, reversible aggregation structures, or equilibrium with soluble monomeric forms and β_2_GPI non-stable dimers, that can be stabilized by covalent intermolecular binding (36). The covalent dimerization associates with thrombotic and inflammatory effects and can be produced either by the cleavage of plasmin or elastase or by the immobilization of the protein molecules in dimerization prone conformation induced by binding to antiphospholipid autoantibodies or negative surfaces. Direct and indirect interactions were described, as well as several complex loops through signaling pathways that are also sensitive to lipoprotein profiles, pH, and salt concentration, which modulate the biochemical effects of β_2_GPI.

Importantly, stable dimerization occurs after a two-step mechanism: protein cleavage and disulfide bond reduction. The stable dimer can also be formed from clipped β_2_GPI either *in vivo* or under long-term storage. It was previously described to be formed by β_2_GPI during purification procedures, but we could identify this form in human plasma after long-term storage, implying plasmin modification as one main source (13). These events are not concomitantly favored in most inflammatory environments *in vivo*. One exception is the situation in which plasmin or elastase activation proceeds during an intermittent or recurrent microcirculatory collapse. Otherwise, an extreme resort to free nitric oxide from protein thiols in low oxygen pressures, the recurrent hypoxia provides the conditions for dimerization of nicked β_2_GPI molecules that remain bound to endothelial surfaces. However, in the presence of higher oxygen concentrations or important recurrence, extensive tissue remodeling ensues the inflammatory and thromboembolic signaling discharge of the resulting endothelial activation, as can be seen in primary antiphospholipid autoimmunity disease.

The covalent dimer was shown to inhibit proliferation and endothelial differentiation. Free forms in ischemic blood may also be cross-linked on the surface of apoptotic remains, oxidized lipoproteins, and cell debris, signaling for non-inflammatory cell removal of these particles (37). During inflammation and tissue repair, several mechanisms cooperate for β_2_GPI distribution, *in vivo* (13). Autoimmune fetal losses triggered by anti-β_2_GPI antibodies may be associated with inflammatory thrombosis and inappropriate angiogenesis.

Moreover, the affinity of β_2_GPI with different substrates is modified by the conformational change that results from dimerization. There are still drawbacks relative to reversible protein conformation changes favoring cleavage and dimerization. Protein conformation may undergo a partially reversible conformation change in response to subtle effects triggered by surfaces and microenvironment composition. Conformation changes favor monomer aggregation and nonstable dimer formation, besides switching between closed and open (J shaped) monomeric forms (6). Thus, given the ability of β_2_GPI to change its structure and adopt new conformations, this protein acts in many different situations and targets several different protein cascades. It is not yet known whether this diversity is directly related to the ability of β_2_GPI to dimerize after conformation change (6).

The multiplicity of β_2_GPI effects reiterates intracellular signaling pathways as likely biochemical targets for its function. There have been some reports on the native β_2_GPI modulation of Akt and MAPKs biochemical signaling pathways, more specifically the ERK 1/2 family proteins, JNK, and p38, but it remains unknown whether and how dimer formation and stabilization bear any relevance to these events (20,21).

In the current study, the *in vivo* effects of two different β_2_GPI purified subfractions confirmed this protein's impact on angiogenesis, suggesting β_2_GPI played an important role in inflammatory-related endothelial cell growth regulation, as previously described by Gomes et al. (38). It is estimated that endothelial cells can cleave β_2_GPI monomers and promote their dimerization. This interaction requires a paracrine/autocrine route in which an endothelial switch of divergent angiogenesis events may occur, as previously observed *in vitro* by Machado et al. (11).

## References

[1] Mifsud W, Sebire NJ. Placental pathology in early-onset and late-onset fetal growth restriction. Fetal Diagn Ther 2014; 36: 117–128, doi: 10.1159/000359969. 10.1159/000359969 24577279

[2] Shibuya M. Vascular endothelial growth factor and its receptor system: physiological functions in angiogenesis and pathological roles in various diseases. J. Biochem 2013; 153: 13–19, doi: 10.1093/jb/mvs136. 10.1093/jb/mvs136 PMC3528006 23172303

[3] Sakai T, Balasubramanian K, Maiti S, Halder JB, Schroit AJ. Plasmin-cleaved beta-2-glycoprotein 1 is an inhibitor of angiogenesis. Am J Pathol 2007; 171: 1659–1669, doi: 10.2353/ajpath.2007.070146. 10.2353/ajpath.2007.070146 PMC2043526 17872974

[4] Yu P, Passan FH, Yu DM, Denyer G, Krilis SA. Beta2-glycoprotein I inhibits vascular endothelial growth factor and basic fibroblast growth factor induced angiogenesis through its amino terminal domain. J Thromb Haemost 2008; 6: 1215–1223, doi: 10.1111/j.1538-7836.2008.03000.x. 10.1111/j.1538-7836.2008.03000.x 18452581

[5] Nakagawa H, Yasuda S, Matsuura E, Kobayashi K, Ieko M, Kataoka H, et al. Nicked {beta}2-glycoprotein I binds angiostatin 4.5 (plasminogen kringle 1-5) and attenuates its anti-angiogenic property. Blood 2009; 114: 2553–2559, doi: 10.1182/blood-2008-12-190629. 10.1182/blood-2008-12-190629 19625706

[6] McDonnell T, Wincup C, Buchholz I, Pericleous C, Giles I, Ripoll V, et al. The role of beta-2-glycoprotein I in health and disease associating structure with function: more than just APS. Blood Rev 2020; 39: 100610, doi: 10.1016/j.blre.2019.100610. 10.1016/j.blre.2019.100610 PMC7014586 31471128

[7] Homolo Gene. HomoloGene:26. Gene conserved in Amniota.https://www.ncbi.nlm.nih.gov/homologene/26. Accessed June 25, 2020.

[8] Miyakis S, Robertson SA, Krilis SA. Beta 2 glycoprotein I and its role antiphospholipid syndrome-lessons from knockout mice. Clin Immunol 2004; 112: 136–143, doi: 10.1016/j.clim.2004.02.014. 10.1016/j.clim.2004.02.014 15240156

[9] Haselgrübler R, Stübl F, Essl K, Iken M, Schröder K, Weghuber J. Gluc-HET, a complementary chick embryo model for the characterization of antidiabetic compounds. PLoS One 2017; 12: e0182788, doi: 10.1371/journal.pone.0182788. 10.1371/journal.pone.0182788 PMC5544204 28777818

[10] Schreiber K, Hunt BJ. Managing antiphospholipid syndrome in pregnancy. Thromb Res 2019; 181: S41–S46, doi: 10.1016/S0049-3848(19)30366-4. 10.1016/S0049-3848(19)30366-4 31477227

[11] Machado C, Nicot ME, Stella CN, Vaz S, Prado C, Maria DA, et al. Digital image processing assessment of the differential in vitro antiangiogenic effects of dimeric and monomeric beta2-glycoprotein I. J Cytol Histol 2013; 4: 187, doi: 10.4172/2157-7099.1000187.

[12] Polz E, Wurm H, Kostner GM. Investigations on ß2-glycoprotein I in the rat: isolation from serum and demonstration in lipoprotein density fractions. Int J Biochem 1980; 11: 265–570, doi: 10.1016/0020-711X(80)90229-3. 10.1016/0020-711x(80)90229-3 7389984

[13] Stella CN, Gomes LF. Monoclonal antibodies anti-beta2-glycoprotein I: production, characterization, analytical applications 1st ed. Saarbrücken: Lambert Academic Publishing; 2013.

[14] Ribatti D, Vacca A, Roncali L, Dammacco F. The chick embryo chorioallantoic membrane as a model for in vivo research on angiogenesis. Int J Dev Biol 1996; 40: 1189–1197. 9032025

[15] Pereira-Lopes JEF, Barbosa MR, Stella CN, Santos WA, Pereira EM, Nogueira-Neto J, et al. In vivo anti-angiogenic effects further support the promise of the antineoplasic activity of methyl jasmonate. Braz J Biol 2010; 70: 443–449, doi: 10.1590/S1519-69842010000200029. 10.1590/s1519-69842010000200029 20549071

[16] Ojala T, Pietikäinen M, Harwood D. A comparative study of texture measures with classification based on featured distributions. Pattern Recognit 1996; 29: 51–59, doi: 10.1016/0031-3203(95)00067-4.

[17] Ojala T, Pietikäinen M, Mäenpää T. Grayscale and rotation invariant texture classification with local binary patterns. In:Computer Vision - ECCV 2000. ECCV 2000. Lecture Notes in Computer Science, vol 1842. Springer, Berlin, Heidelberg. doi: 10.1007/3-540-45054-8_27.

[18] Varga Z, Flammer AJ, Steiger P, Haberecker M, Andermatt R, Zinkernagel AS, et al. Endothelial cell infection and endothelitis in COVID-19. Lancet 2020; 395: 1417–1418, doi: 10.1016/S0140-6736(20)30937-5. 10.1016/S0140-6736(20)30937-5 PMC7172722 32325026

[19] Pierangeli SS, Chen PP, Raschi E, Scurati S, Grossi C, Borghi MO, et al. Antiphospholipid antibodies, and the antiphospholipid syndrome: pathogenic mechanisms. Semin Thromb Hemost 2008; 34: 236–250, doi: 10.1055/s-0028-1082267. 10.1055/s-0028-1082267 18720303

[20] Regan CP, Li W, Boucher DM, Spatz S, Su MS, Kuida K. Erk5 null mice display multiple extraembryonic vascular and embryonic cardiovascular defects. Proc Natl Acad Sci USA 2002; 99: 9248–9253, doi: 10.1073/pnas.142293999. 10.1073/pnas.142293999 PMC123126 12093914

[21] Yang J, Boerm M, McCarty M, Bucana C, Fidler IJ, Yang YZJ, et al. Mekk3 is essential for early embryonic cardiovascular development. Nat Genet 2000; 24: 309–313, doi: 10.1038/73550. 10.1038/73550 10700190

[22] Giannakopoulos B, Mirarabshahi P, Krilis SA. New insights into the biology and pathobiology of beta2-glycoprotein I. Curr Rheumatol Rep 2011; 13: 90–95, doi: 10.1007/s11926-010-0151-9. 10.1007/s11926-010-0151-9 21089000

[23] Chiu WC, Chiou TJ, Chung MJ, Chiang AN. β2-Glycoprotein I inhibits vascular endothelial growth factor-induced angiogenesis by suppressing the phosphorylation of extracellular signal-regulated kinase 1/2, Akt, and endothelial nitric oxide synthase. PLoS One 2016; 11: e0161950, doi: 10.1371/journal.pone.0161950. 10.1371/journal.pone.0161950 PMC5006999 27579889

[24] Shen L, Azmi NU, Tan XW, Yasuda S, Wahyuningsih AT, Inagaki J, et al. Mutants of β2-glycoprotein I: their features and potent applications. Best Pract Res Clin Rheumatol 2018; 32: 572–590, doi: 10.1016/j.berh.2019.01.007. 10.1016/j.berh.2019.01.007 31174826

[25] Beecken WD, Ringel EM, Babica J, Oppermann E, Jonas D, Blaheta RA. Plasmin-clipped beta(2)-glycoprotein-I inhibits endothelial cell growth by down-regulating cyclin A, B, and D1 and up-regulating p21 and p27. Cancer Lett 2010; 296: 160–167, doi: 10.1016/j.canlet.2010.04.010. 10.1016/j.canlet.2010.04.010 20435405

[26] Wang QQ, Zhou SJ, Meng ZX, Wang J, Chen R, Lv L, et al. Domain I--IV of β2-glycoprotein I inhibits advanced glycation end product-induced angiogenesis by down-regulating vascular endothelial growth factor 2 signaling. Mol Med Rep 2015; 11: 2167–2172, doi: 10.3892/mmr.2014.2970. 10.3892/mmr.2014.2970 25405610

[27] Matschke K, Silva-Azevedo L, Hlushchuk R, Djonov V, Baum O. Annexins as cell-type-specific markers in the developing chicken chorioallantoic membrane. Cell Tissue Res 2006; 323: 395–404, doi: 10.1007/s00441-005-0112-1. 10.1007/s00441-005-0112-1 16344946

[28] Sharma MC, Sharma M. The role of annexin II in angiogenesis and tumor progression: a potential therapeutic target. Curr Pharm Des 2007; 13: 3568–3575, doi: 10.2174/138161207782794167. 10.2174/138161207782794167 18220793

[29] Su SC, Maxwell SA, Bayless KJ. Annexin 2 regulates endothelial morphogenesis by controlling AKT activation and junctional integrity. J Biol Chem 2010; 285: 40624–40634, doi: 10.1074/jbc.M110.157271. 10.1074/jbc.M110.157271 PMC3003361 20947498

[30] Chong DC, Koo Y, Xu K, Fu S, Cleaver O. Stepwise arteriovenous fate acquisition during mammalian vasculogenesis. Dev Dyn 2011; 240: 2153–2165, doi: 10.1002/dvdy.22706. 10.1002/dvdy.22706 PMC3192916 21793101

[31] Lawson ND, Scheer N, Pham VN, Kim CH, Chitnis AB, Campos-Ortega JA, et al. Notch signaling is required for arterial-venous differentiation during embryonic vascular development. Development 2001; 128: 3675–3683. 10.1242/dev.128.19.3675 11585794

[32] Ioannou Y. The Michael Mason prize: pathogenic antiphospholipid antibodies, stressed out antigens, and the deployment of decoys. Rheumatol 2012; 51: 32–36, doi: 10.1093/rheumatology/ker353. 10.1093/rheumatology/ker353 22120465

[33] Ioannou Y, Zhang JY, Passam FH, Rahgozar S, Qi JC, Giannakopoulos B, et al. Naturally occurring free thiols within beta2‐glycoprotein I in vivo: nitrosylation, redox modification by endothelial cells, and regulation of oxidative stress‐induced cell injury. Blood 2010; 116: 1961–1970, doi: 10.1182/blood-2009-04-215335. 10.1182/blood-2009-04-215335 20551379

[34] Meroni PL, Tincani A, Sepp N, Raschi E, Testoni C, Corsini E, et al. Endothelium and the brain in CNS lupus. Lupus 2003; 12: 919–928, doi: 10.1191/0961203303lu503oa. 10.1191/0961203303lu503oa 14714912

[35] Chighizola CB, Pregnolato F, Andreoli L, Bodio C, Cesana L, Comerio C, et al. Beyond thrombosis: anti‐β_2_GPI domain 1 antibodies identify late pregnancy morbidity in anti‐phospholipid syndrome. J Autoimmun 2018; 90: 76–83, doi: 10.1016/j.jaut.2018.02.002. 10.1016/j.jaut.2018.02.002 29454510

[36] Averna M, Paravizzini G, Marino G, Emmanuele G, Cefalù AB, Magro G, et al. β_2_-glycoprotein I is growth regulated and plays a role as a survival factor for hepatocytes. Int J Biochem Cell Biol 2004; 36: 1297–1305, doi: 10.1016/j.biocel.2003.10.017. 10.1016/j.biocel.2003.10.017 15109573

[37] Gomes LF, Gonçalves LM, Fonseca FLA, Celli CM, Videla LA, Chaimovich H, et al. Beta2-glycoprotein I (apolipoprotein H) modulates uptake and endocytosis associated chemiluminescence in rat Kupffer cells. Free Radic Res 2002; 36: 741–747, doi: 10.1080/10715760290032548. 10.1080/10715760290032548 12180124

[38] Gomes LF, Knox PR, Simon-Giavarotti KA, Junqueira VB, Sans J, Videla LA. Beta2-Glycoprotein I inhibition of mouse Kupffer cells respiratory burst depends on the liver architecture. Comp Hepatol 2004; 3: 1: S43, doi: 10.1186/1476-5926-2-S1-S43. 10.1186/1476-5926-2-S1-S43 PMC2409444 14960195

